# The potential of AB-free kava in enabling tobacco cessation via management of abstinence-related stress and insomnia: study protocol for a randomized clinical trial

**DOI:** 10.1186/s12906-024-04722-9

**Published:** 2024-12-21

**Authors:** Chengguo Xing, John Malaty, Melissa Bou Malham, Frank A. Orlando, Allison Lynch, Zhiguang Huo, Magda François, Roberto Firpi-Morell, Carla L. Fisher, Demetra D. Christou, Ramzi G. Salloum

**Affiliations:** 1 College of Pharmacy, University of Florida, Gainesville, FL USA; 2 College of Medicine, University of Florida, Gainesville, FL USA; 3 College of Health and Human Performance, University of Florida, Gainesville, FL USA

**Keywords:** Randomized controlled trial, Stress and insomnia, Smoking Cessation, Kava

## Abstract

**Background:**

As the primary cause of various preventable illnesses, smoking results in approximately five million premature deaths each year in the US and a multitude of adults living with serious illness. The majority of smokers know the health risks associated with smoking and intend to quit. However, quitting is very difficult partly because of insomnia and stress associated with it. Current tobacco cessation medications are not designed to address these problems, which may have contributed to their limited success in enabling cessation. Novel interventions are thus urgently needed to enhance success rates in tobacco cessation. Based on its historical usage and our preliminary data, kava is such a candidate. Kava, customarily enjoyed by South Pacific Islanders, is known for its relaxing effects, stress-relieving properties, and ability to enhance sleep. In the US, it is marketed and distributed as a dietary supplement due to its recognized calming properties. A pilot trial was performed among active smokers with a one-week ingestion of a kava supplement. The results for the first-time revealed kava’s potential in enabling tobacco cessation with effects on a panel of biological signatures. The primary goal of this trial is to replicate kava’s effects on the biological signatures of tobacco use, stress, and sleep in addition to its compliance and safety among those who smoke.

**Methods:**

A double-blind randomized placebo controlled two-arm trial will enroll 76 smokers with intention to quit, who will consume AB-free kava at a dietary supplement dose or placebo, 3 times per day for 4 weeks with two follow-ups.

**Discussion:**

The study will (1) monitor the adherence to and safety of AB-free kava consumption among smokers and evaluate changes in smoking habits, and (2) quantify a panel of non-invasive translatable biomarkers to objectively evaluate AB-free kava’s holistic effects on biological signatures associated with tobacco use, stress, and sleep. We hypothesize that AB-free kava is a novel and promising intervention to facilitate tobacco cessation via its holistic effects associated with managing stress and insomnia during abstinence. If the results from this study support our hypothesis, kava could emerge as an affordable and accessible dietary supplement candidate for tobacco cessation.

**Trial Registration:**

registered on 04/14/2023 in ClinicalTrials.gov with the identifier NCT05814055.

**Supplementary Information:**

The online version contains supplementary material available at 10.1186/s12906-024-04722-9.

## Introduction

### Background and rationale

There are approximately 34 million adults who smoke in the US [1, 2] with scant evidence suggesting a significant decrease in this number in the foreseeable future [3]. Tobacco consumption remains the primary contributor to various preventable illnesses, and roughly half of smokers are at risk of dying due to smoking-related complications if they do not quit [1]. Given that ~ 70% of adult smokers want to stop smoking and over 50% of them make a quit attempt annually [4], there’s a potential to greatly alleviate this health issue by enhancing the effectiveness of tobacco cessation methods. Importantly, two thirds of those smokers who plan to quit have shown interest in exploring complementary approaches to facilitating tobacco cessation methods [1], an aspect that is currently lacking. Effective complementary tobacco cessation interventions, therefore, need to be developed to support smokers achieve cessation and reduce the risk of associated diseases.

The first-line medications to facilitate tobacco cessation include nicotine replacement therapy, bupropion, and varenicline. Such medications typically result in moderate success rates, but patients suffer from significant relapses [5]. Some of them are also linked to various adverse side effects, which may have contributed to their limited success in tobacco cessation. One common side effect is sleep disturbance including insomnia [6]. It has been even proposed that adjunctive treatment targeting sleep disturbance may improve abstinence rates [6, 7]. 

Kava is historically known to be a safe beverage and is renowned for enhancing sleep and promoting relaxation with daily dosages ranging between 750 and 8,000 mg kavalactones [8–12]. Upon reviewing safety data on kava, the WHO and other organizations have determined that kava’s risk of liver toxicity is exceptionally rare, if it indeed exists, with less than 0.3 cases per one million daily doses, particularly when using the appropriate cultivars and preparations [13]. Kava therefore has always been accessible in the US market in the form of a dietary supplement. Kava’s hepatotoxic risk may be associated with ingredients enriched in the anxiolytic preparations because kava has been traditionally consumed for centuries and its daily kavalactone dose is significantly (3–100 times) higher than its anxiolytic and dietary supplement regimens (120–280 mg kavalactones daily) [8–12, 14]. One possible reason could be the elevated concentration of flavokavains A and B present in the anxiolytic variant, which are roughly 100 times higher compared to traditional kava [15]. Flavokavains A and B are found in significantly higher quantities in kava cultivars of low-quality compared to noble cultivars [16]. These compounds exhibit the highest cytotoxicity among all substances found in kava [15–17] and have caused liver toxicity in laboratory animals [18, 19]. These findings indicate that flavokavains A and B play a major role in kava’s risk of liver toxicity.

Thorne developed a kava formulation (namely AB-free kava), devoid of flavokavain A and B, and designed it to closely resemble the traditional kava preparation. AB-free kava has rigorous Chemistry Manufacturing and Controls (CMC) documentation, high quality control and quality assurance, and an enabled IND status (142838) with an ongoing double-blind randomized placebo-controlled trial among patients with generalized anxiety disorder (NCT03843502) using the same regimen as proposed in this application. This formula has also demonstrated outstanding safety profiles in several animal models [20–23]. The pharmacokinetics of six major kavalactones in AB-free kava has been characterized among healthy participants [24], indicating that a three times daily regimen offers better kavalactone exposure.

Based on its relaxing and sleep-enhancing properties [25–28], kava holds promise in assisting cessation efforts and could potentially offer great efficacy. Its dietary supplement status may make it more acceptable to smokers as well [29]. These potential benefits were reinforced by the outcomes of our preliminary study investigating the use of kava among smokers [30] where one week of kava use reduced tobacco dependence and, biomarkers on tobacco exposure, stress, and increased biomarkers on sleep.

The main goal is to replicate the effects of kava on biological signatures of tobacco use, stress, and sleep in addition to its compliance and safety among smokers.

### Objectives

This study seeks to fulfill the following goals: (1) To assess adherence to AB-free kava and determine any possible concerns; (2) To investigate whether AB-free kava could aid in tobacco cessation, using participant self-report questionnaires alongside a decrease in urinary total nicotine equivalents (TNE), which assesses cotinine, nicotine, and 3-hydroxycotinine and their conjugates, as well as nicotine-*N*-oxide; (3) To examine if AB-free kava can decrease stress and improve sleep – standard questionnaires and associated biomarkers (plasma PRKACA, plasma cortisol, urinary total cortisol equivalents (TCE), and urinary N-acetyl serotonin (NAS)). (4) To use a wrist wearable device to objectively monitor parameters related to sleep, which complement self-reported sleep measures. An additional exploratory objective is to examine the sociocultural context to better understand variables that could influence participants’ enrollment and willingness to engage in kava use as a dietary supplement in tobacco cessation.

### Hypothesis

Our hypothesis proposes that AB-free kava, formulated correctly, is a novel, promising intervention that facilitates tobacco abstinence by addressing stress and insomnia associated with cessation in a holistic manner. To test this hypothesis, we plan to perform a two-arm trial involving 76 smokers intending to quit, using a double-blind randomized placebo-controlled design. Participants will either receive AB-free kava or a placebo during the 4-week treatment duration.

### Aims

This trial has two goals: (1) To record compliance with AB-free kava, detect any possible problems, and characterize its effects on smoking, stress, and sleep associated behaviors. (2) To quantify a panel of non-invasive clinically translatable biological variables to objectively evaluate AB-free kava’s holistic effects on biological signatures associated with tobacco use, stress, and sleep.

### Trial design

To test our hypothesis, we present a double-blind randomized placebo-controlled longitudinal trial that includes 4 weeks of intervention with follow-up visits. The study team will enroll smokers with intention to quit and randomize them into one of two groups. Patients that do not want to quit will be referred to other smoking cessation studies or to the Tobacco Free Florida, a state program.

After randomization, we will have two groups (AB-free kava vs. placebo) taking one capsule 3 times daily of their respective treatment. One of the groups will receive capsules that contain the placebo, whereas the second group will be provided with AB-free kava capsules. Sample collections and safety monitoring will occur at Week 0, 1, 2, 4, 8 and 12.

Week 8 and 12 are follow-up visits designed to (1) determine whether AB-free kava’s potential is sustainable or not; and (2) explore adverse effects that may appear at a later time. Follow-up visits are essential for both participants and future development. The regimen proposed herein remains identical to that in the pilot study [30], backed by our recent pharmacokinetic findings indicating that AB-free kava’s ingredients have a half-life of not more than 3 h.

The proposed 4-week intervention exposure is anticipated to give ample data about the adherence, effectiveness, and safety of AB-free kava, with the aim of mitigating potential risks for the participants.

After completing the study, study coordinators will refer participants to Tobacco Free Florida with the aim of enhancing their likelihood of quitting tobacco consumption.

## Methods: participants, interventions, and outcomes

The SPIRIT reporting guidelines and SPIRIT figure were used to create this manuscript [31]. Our methods section closely resembles but is not identical to that of the published protocol on AB-free kava’s effect on decreasing tobacco-associated lung cancer risk [32]. 

### Study setting

The study will take place within clinics of University of Florida (UF) Health Family Medicine. Clinic staff members from the UF Health Family Medicine Main Street Clinic and Springhill Clinic, under the supervision of medical doctors, will identify possible participants and introduce the overall goal of the study, typically during the regular clinic visit of the prospective participants.

### Study setting justification

UF Health Family Medicine Main Street Clinic and Springhill sites are chosen for the following reasons: (a) over 1700 adult smokers visited the Main Street Clinic last year for annual checkup, > 50% of whom smoke at least 10 cigarettes/day. Although our exclusion criteria are stringent (detailed below), we expect at least 15–20% recruitment rate given the high success to recruit participants in family clinics for tobacco trials; (b) successful track record of recruiting smokers to support clinical research, including collaborations between our co-investigators who have previously recruited 120 adult smokers from the same clinic in 4.5 months (NCT03836573); (c) electronic health record information of patients in the clinics will help identify eligible participants – those without previous liver diseases; and (d) most importantly, the clinical expertise, resources, and facilities will minimize potential adverse effects in this trial. The Springhill site has a similar patient population and clinical research structure. We propose two sites to maximize enrollment.

### Eligibility criteria

In this study, we have utilized the same eligibility criteria as our previously published paper “Reducing tobacco-associated lung cancer risk: a study protocol for a randomized clinical trial of AB-free kava” with one notable exception: participants are now required to express a desire to quit. This modification reflects an evolving emphasis on participant motivation and readiness to engage in cessation efforts, which was not a prerequisite in our earlier investigation. By incorporating this criterion, we aim to enhance the relevance and applicability of our findings to current clinical practices. This adjustment ensures that our study maintains methodological rigor while accommodating advancements in the field, ultimately contributing to a more nuanced understanding of smoking cessation interventions.

By adhering to established criteria, we aimed to maintain methodological continuity and facilitate direct comparisons with previous findings. This approach not only strengthens the coherence of our study design but also underscores the reliability and validity of our results within the context of our ongoing research program.

### Intervention description

The trial will require resources from both the UF Family Medicine Clinics and UF Consent2Share to assist in the recruitment of active smokers intending to quit, who are otherwise healthy. These participants will be assigned randomly to either the placebo group or the experimental group receiving AB-free kava (225 mg dose), undergoing a four-week exposure period with a total of six visits (Week 0, 1, 2, 4, 8, and 12). The primary objectives of these visits are to assess adherence and pinpoint any potential challenges related to the use of AB-free kava. Moreover, this study seeks to investigate how AB-free kava affects tobacco use and dependence, stress levels, sleep patterns, and associated indicators among study enrollees.

**Fig. 1:**
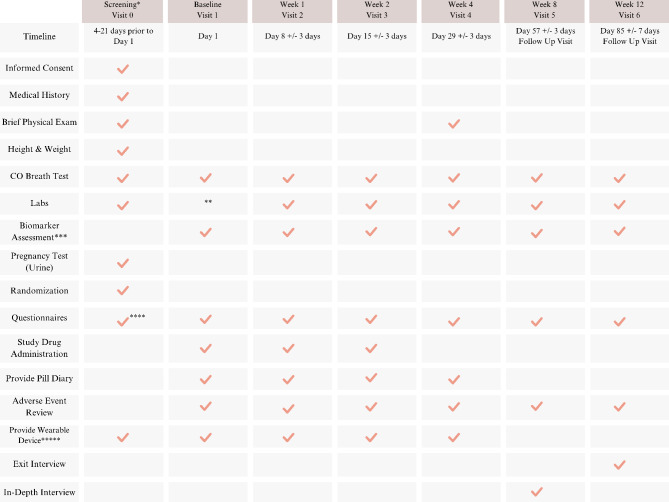
Participant timeline that includes an AB-free Kava intervention, followed by follow-up sessions. *Legend:* *: Screening procedures conducted according to established medical standards during the designated screening period can be administered before formal consent is obtained. **: If the baseline visit is within 7 days of the screening, there is no need to redraw blood. Labs are listed in the table below. ***: Blood and urine markers. ****: Only ASQ questionnaire is administered at screening. *****: GTR3X + will be provided to participants. The participants will wear the device for 7 days after visits 0, 1, and 2, and 7 days prior to visit. They will also be instructed to refrain from wearing the device following visit 3

A total of three capsules of placebo or AB-free kava is administered by mouth daily. Participants will be instructed to take one capsule in the morning, at noon, and in the evening, at approximately the same time each day. Each AB-free kava capsule contains 75 mg of kavalactones, resulting in daily regimen of 225 mg. The dosage will remain unmodified, and in case of any adverse effect (detailed in a later section), usage will be stopped. Permission to conduct this study will be obtained from the UF IRB prior to start. The content of each visit is outlined in the participant timeline depicted in Fig. 1.

Potential participants will be identified and referred to the study coordinators by the clinic staff members. Upon referral, the coordinator will conduct the pre-screening interview. During the interview, the study and its potential benefits and risks will be explained. The study coordinator will schedule interested candidates that meet eligibility criteria for a pre-screening visit. At that visit, study coordinators will facilitate the process of informed consent for potential participants. This involves ensuring individuals receive comprehensive information about the study, and their rights as participants. The study coordinator will also emphasize the importance of limiting alcohol consumption and avoiding any medications containing acetaminophen. Participants will then verbalize understanding that their participation is voluntary, and they have the option to withdraw without facing implications regarding their current or future healthcare. They will also be made aware that they may be withdrawn by the study team if they exhibit non-adherence to the protocol, fail to limit alcohol intake, or experience adverse events, among other reasons explained later.

After allowing them to review the informed consent and ask any questions, they will sign the consent form electronically in the highly secure and HIPAA-compliant web-based application, Research Electronic Data Capture (REDCap). Study coordinators will inform the candidates that they will receive a $50 gift card/visit at Visit 1–6 that will total up to $300 if they attend all visits. This $300 amount does not include the optional $25 that selected subjects may receive for consenting to complete the post-treatment interview at Visit 4 (or whenever they stop treatment). If subjects complete all visits and do the optional post-treatment interview, then they can receive a total of $325.

Participants will then take a Carbon Monoxide (CO) breath test to assess smoking status. Blood will be collected to determine whether ALT, AST, ALP, and total bilirubin levels fall within normal ranges. A pregnancy spot urine test will also be done for females of childbearing potential. Once eligibility criteria are confirmed, qualified participants will be enrolled into this study and assigned randomly to either of the study groups. Visit 1 will also be set up within 4 to 21 days later. The research coordinator will provide the participant with a GT3X + accelerometer wearable device and will instruct them to wear it for seven days after the first three visits at Visit 0, Visit 1, and Visit 2. The research coordinator will also instruct participant to wear the device 7 days prior to Visit 4. The wearable device will use a micro-electro-mechanical system (MEMS) based accelerometer and an ambient light sensor to measure physical activity and sleep. The device will be programmed to begin recording at midnight after their visit. The study coordinator will provide instruction on the use of the wearable device, including demonstration on how to correctly wear the device.

**Fig. 2:**
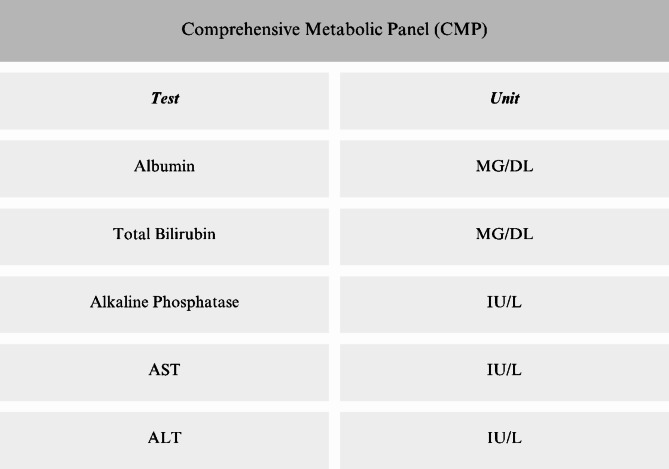
List of CMP lab values

Each of the six visits, following the screening visit, will last around 30–60 min. These visits will involve blood tests for ALT, AST, ALP, total bilirubin levels, a breath CO test, and the administration of questionnaires. Figure 2 details the contents of the Comprehensive Metabolic Panel lab test along with their respective units. A10 mL blood sample will also be collected onsite, along with a 24-hour urine collection, for biomarker analysis. The participants will also be given the medication in a bottle that has written instructions and a pill diary to keep track of their medication doses and note the corresponding times. This tool will be useful for comparing the number of pills returned in the bottle to the information recorded in the pill diary. Additionally, they will be provided with materials for the upcoming urine collection. Participants will have a continuous access to the study coordinator, fostering a strong rapport to encourage their return. Additionally, they will be closely monitored throughout their participation, and study visits will be confirmed via reminder phone calls. Upon completion of each visit, participants will receive a debit card loaded with funds, which will be reloaded at every subsequent visit. The study coordinators will consistently employ respectful language and demonstrate empathy when communicating with the participants.

During Visit 1–4, the data stored on the GT3X + wearable device will be downloaded by the study coordinator. At Visit 4, the GT3X + wearable device will be collected from the participants.

At the conclusion of the study (Visit 6) or upon a participant’s withdrawal from the study, they will undergo an exit interview consisting of open-ended questions to gather their feedback on the trial.

At the end of the treatment, Visit 4, or at the time a participant withdraws from the study, some participants will be offered an opportunity to complete a post-treatment interview. If they choose to participate, they will receive an additional $25 to the $50 they are already being given for their attendance at that visit. Participants who are invited to complete the post-treatment interview will be purposively sought to ensure comparable subgroups of racial and ethnic minorities and white participants. By recruiting these subgroups, we can explore cultural similarities and differences in their experiences. We expect to recruit a total of 25 participants to complete the post-treatment interview to ensure thematic saturation. However, data collection and analysis will be concurrent to ensure saturation is reached. This interview will help to identify facilitators and barriers to trial enrollment and retention.

Due to the established rapport and trust, the study coordinator will conduct thorough, semi-structured interviews either via phone or Zoom [33]. Dr. Fisher (co-I), a qualitative methodology expert in health behavior intervention development, will provide oversight on data collection and analysis. She will also develop the semi-structured script and train the coordinator on in-depth interviewing. Interview questions will explore participants’ experiences with kava to identify critical factors to promoting future trial enrollment, adherence, and sustained healthy behavior change [34, 35]. Questions will explore facilitators and barriers to enrollment and retention as well as sociocultural factors informing participants’ health behavior and ongoing willingness to engage in kava use. These include exploring the influential role of cultural norms or beliefs, experiences within their family system, and prior use or beliefs about kava. The research coordinator will audio record the interviews and will send the files to be transcribed professionally. The transcriptions of interviews are the qualitative data that will be thematically analyzed by a qualitative research scientist. Without audio files or transcriptions, there will be no reliable data. This approach conforms with standard and best practices in qualitative methodology.

### Outcomes

The primary endpoint is to evaluate participants’ compliance to AB-free kava. Such information is critical for data interpretation and future clinical trial improvement. We will evaluate trial compliance using the same three methods employed during the pilot trial: [30] (i) participants will report any missed doses via their pill diaries; (ii) the UF IDS pharmacy will count returned pills; and (iii) and the study team will analyze participants’ urine for the presence of dihydromethysticin (DHM) during kava ingestion, as DHM is specific to kava [36]. In combination with the exit interview, the results will provide knowledge of the practicality of using AB-free kava and potential issues with current treatment regimen among smokers.

Another study aim is to investigate if AB-free kava can help patients quit smoking, reduce stress, and enhance sleep quality. Tobacco dependence will be determined using questionnaires, inquiring about participants’ smoking urges. Tobacco use will be assessed using measurement of urinary TNE. Stress reduction will be studied using perceived stress questions and stress biomarkers such as plasma cortisol, PRKACA, and urinary TCE. Sleep improvement will be determined using sleep quality questionnaires, wearable accelerometer measurements, and sleep biomarkers (urinary 6-hydroxymelatonin and urinary NAS). Assessments will be made on the variations in participants’ results and responses in comparison to their baseline data.

### Participant timeline

### Sample size

Our targeted accrual goal is 76 active smokers. Based on our previous enrolling experience at the UF Health Family Medicine Clinic and our rather strict eligibility criteria, the enrollment is expected to be 1.5–2.5 years with the study duration of each participant to be approximately 12 weeks.

The sample size was determined based on our primary biological signature endpoint data (TNE and PRKACA) from our pilot results. Using the pilot result of TNE as an example, the ratio of geometric mean of TNE after one week kava treatment is 0.68 with a standard error of 0.06. By taking into consideration that the placebo group demonstrates 15% of the treatment impact, a figure widely agreed upon in existing literature [37, 38], and apply the identical standard error as observed in the kava treatment group, the standardized effect size (Cohen’s d) equals d = 1.01. Similarly, the Cohen’s d for PRKACA is 0.89. Since in our proposed study, we will repeatedly measure these biomarkers in each participant, we further assume the same Kava effect from week one through week four. By (i) varying within-participant correlation rho = 0, 0.2, 0.4, 0.6, and 0.8; (ii) at Bonferroni-corrected alpha level 2.5% (5%/2), and (iii) using a linear mixed model, 60 participants (30 per group) are needed to have at least 90% power for both TNE and PRKACA. Additionally, supposing an 80% retention rate, consistent with rates in comparable studies [39], tour plan entails recruiting 76 participants, with 38 assigned to each group.

The research team will exclude pregnant women from this study due to the proposed method involving AB-free kava to help reduce tobacco use by managing stress. This decision is based on the anticipation that pregnancy may induce substantial alterations in mental state and hormonal physiology. These discrepancies are likely to impede the primary objective of this early-phase clinical investigation by weakening the justification for determining the sample size.

### Recruitment

We intend to reach our target sample size through several strategies. Firstly, we will engage the clinic staff, ensuring they are well-informed about the study and addressing any questions they may have. Secondly, we will utilize UF Consent2Share to disseminate our study and connect with a larger number of patients who visit UF Health clinics. Additionally, consistent reminders and effective communication with clinic staff will be maintained throughout the study. Flyers promoting the study will be distributed at the clinic site to motivate patients to participate. Our study coordinators will focus on establishing strong rapport with patients to enhance retention rates. Lastly, participants’ involvement will be compensated through the distribution of debit cards.

## Methods: assignment of interventions

### Randomization

A 1:1 allocation scheme will be used to randomize eligible participants into either the AB-free kava group or the placebo group. The randomization process will be stratified by a participant’s age, gender and their daily cigarette consumption (heavy vs. light.) Daily cigarette consumption is classified as heavy when an individual consumes 25 or more cigarettes per day [40]. Furthermore, with every set of four participants categorized together, two we will be randomly assigned to each of the two groups. Employing this approach of stratified block randomization ensures an equitable distribution of sample size across the groups throughout the study period.

Dr. Huo (Biostatistician) will implement this randomization within the REDCap system. REDCap will use the information entered by the study coordinator to generate a unique label. Dr. Huo will provide the study groups (i.e., placebo group or kava group in a random order) associated with these group labels to the independent UF IDS pharmacy before initiating the study. Participants will receive the supplies for their assigned treatment from a UF IDS pharmacist.

The study is a double-blind trial as such IDS will work autonomously from the study team. IDS will unmask the assignment by providing the study team with the record only upon completion of the study. IDS can perform unblinding in case of medical emergencies. The identity of the individual who unblinded the participant and the rationale for unblinding will be recorded.

### Preparing, dispensing, and recording study supplements

Following randomization, each participant’s supplies will be prepared by the UF Investigational Drug Service (IDS) pharmacy. Participants will use their pill diaries to keep track of any missed doses and return any unused supplies during their subsequent visit for recording and disposal by IDS pharmacy. The IDS pharmacy will maintain an accountability log. Each log comprehensively documents participant-specific details such as participant ID and randomization codes. Additionally, the log will encompass information regarding the provision, return, and disposal of supplements.

AB-free kava and placebo capsules are sourced from Thorne Research Inc. Chemistry, Manufacturing and Controls (CMC) data for these products have undergone revision by the Food and Drug Administration (FDA) with an approved Investigational New Drug (IND, #142838) for a clinical trial involving individuals diagnosed with generalized anxiety disorder (GAD). The IND for a similar study among smokers with no intention to quit has been approved (IND #157256), and this study will be added as an amendment. Our validated LC-MS/MS method has also assessed the capsules for the absence of flavokavains A and B, as well as for the abundance of kavalactones [36]. 

### Data collection

Participant details and survey answers will be managed through a REDCap database and will be securely stored on UF servers. Furthermore, information regarding the biological samples, such as the collection date, any missed samples or doses, and the visit number, will also be documented in REDCap. The study coordinator will transport the blood and urine samples from the clinic to the Xing Lab within one hour of the participant’s visit for storage.

REDCap is a HIPAA-compliant, highly secure, and intuitive-to-use online application used to capture and store clinical research, including questionnaire data from research participants.

Data will be periodically exported to SAS and R data types set for analysis. The integrity of the data will be assessed through descriptive statistics, such as means, standard deviations, frequencies, percentages, and ranges, which are suitable for measurement levels. Additionally, checks will be conducted to identify missing, out-of-range, or implausible values. Biological data will be subject to computerized range and consistency checks as well. Any inaccuracies or discrepancies in data will be addressed in study team meetings. The statistician will generate quarterly statistical summaries and progress for review by all investigators. To maintain patient confidentiality, personal identifiers will be excluded from the dataset. The final set will be made accessible to the research team.

Data will be managed using a data management software program (ATLAS.ti). A thematic analysis will be conducted by a qualitative analyst trained by Fisher, who will oversee analysis and codebook development [41, 42]. To maintain sensitivity to cultural differences, data will be segmented by race and ethnicity and analyzed by group to triangulate findings. An additional coder will be used to validate the final codebook to further promote rigor in analysis [43]. Audio recordings will begin after consent has been obtained and captured using a digital recording device. Audio files (MP3) will be downloaded immediately after the interview onto a secure UF computer. Files will be uploaded for professional transcription with a vendor that has been approved by UF Risk Management with associated approved procedures. Files will be saved as a code instead of patient names. This code will be used on transcriptions as well. A password-protected UF computer, accessible only to the PIs and relevant study personnel, will be used to storing the code-identifier file. Prior to thematic analysis, all transcriptions (i.e., data) will be anonymized to ensure confidentiality of participants. Furthermore, no identifying information will be used in any publications or presentations. Audio files will be destroyed once analysis has been completed. Transcripts will be destroyed once all analyses are completed or within two years once the study period has ended.

### Statistical methods

In preparing this manuscript, we utilized the statistical methods section directly from our previously published protocol paper. This decision was made to ensure accuracy and consistency in the application of statistical techniques relevant to our study, which mirrors those used in the cited research. By adopting these methods, we aimed to maintain methodological rigor and alignment with established practices in our field. This approach not only facilitates reproducibility but also acknowledges the robustness and appropriateness of the statistical methods employed in the original study, thereby enhancing the credibility of our findings within the existing scientific framework.

## Methods: monitoring

### Data monitoring

In accordance with the University of Florida Health Cancer Center’s (UFHCC) data and safety monitoring plan, the PI will submit a Data Integrity and Safety Committee Report to the Data Integrity and Safety Committee (DISC) at scheduled intervals, corresponding to the risk category determined during the initial SRMC CCPSP (Scientific Review and Monitoring Committee Cancer Control and Population Sciences Panel) review, which takes place before initial IRB approval. The PI will personally oversee and manage the conduct of human subjects in alignment with ethical principles and in adherence to federal, state, and local laws, institutional policies, and IRB requirements. The PI’s primary responsibility will be the ongoing monitoring of unanticipated problems, adverse events, unanticipated problems, and other protocol-related concerns.

Continuous monitoring of enrolled participants will be conducted by Drs. Salloum and Firpi (PI and Co-Is), in conjunction with other investigators and IRB. Serious adverse events and unanticipated cases posing risks to participants or others will be promptly reported to the IRB, NIH, and FDA.

The following measures will be implemented for data monitoring:

1. Monitor study progression, adverse events, and data integrity concerns; consider external factor beyond the study when analyzing data, including scientific advancements that might impact participant safety or study ethics; and uphold confidentiality throughout all trial phases.
2. Assess for potential acute or delayed hepatotoxicity by monitoring ALT, AST, ALP, and total bilirubin levels during each visit and comparing them to baseline values. The results, in conjunction with clinical manifestations, will be used to determine whether the drug should be continued or discontinued. Such cases are explained in detail below.
3. Perform routine study audits conducted by the UF DISC (outlined in detail below).

### Possible discomforts and harms

Consuming kava supplements carries minimal potential discomfort and there were no adverse events reported from human kava consumption in the preliminary findings from a prior study. Nevertheless, individuals with a history of liver conditions will be excluded due to the kava’s potential hepatotoxic effect via drug-herb interactions. Additionally, participants’ liver function will be monitored before, during, and after receiving AB-free kava preparations. These preparations lack the potentially hepatotoxic ingredients in kava, flavokavains A and B [18], and are anticipated to have an enhanced safety profile.

Three potential events and outcomes will be considered in the event of elevated liver enzymes:

1. If serum liver enzymes rise above three times the normal level (clinically regarded as a mild elevation), participants will be promptly made aware and scheduled for retesting within the subsequent 48 to 72 h.
2. If liver enzyme levels rise to greater than five time the upper limit of normal (ULN), the consumption of the drug will be immediately ceased, and the participant will be directed for additional clinical assessment and treatment.
3. If there’s a rise in Liver Function Test (LFT) levels exceeding three times the ULN, coupled with hepatotoxicity symptoms, the consumption of the drug will immediately cease, and the participant will be directed for additional evaluation.

Liver enzyme levels will be monitored throughout the treatment period and during the follow-up visits. This duration of monitoring is deemed adequate, even considering the reported cases of hepatotoxicity associated with kava [44]. Participants will also be instructed to limit alcohol intake and avoid acetaminophen containing products for the entire duration of the study. Alcohol consumption will be evaluated through self-reported questionnaires and participants will receive a list of acetaminophen containing drugs. Physicians, including a hepatologists specialized in monitoring liver function, will oversee the safety monitoring of the liver.

No severe adverse effects have been detected in the pilot kava trial and the recent GAD trial. Subjects have the potential to experience other side effects like digestive upset, headache, and dizziness. Because of kava’s properties, its consumption may lead to sedation. Participants will be cautioned against operating heavy machinery and advised to drive carefully within two hours of after using AB-free kava until they understand its effects, despite the low risk associated with the recommended dose.

Blood draws, administered by certified professionals, can also pose minimal risk. There also exists potential confidentiality risks for study participants. Nevertheless, our established procedures designed to protect against such events mitigate these risks to a minimum. Additionally, kava’s potential neurological effects necessitate the utilization of an Ask-Suicide-Screening Questions (ASQ) form to evaluate and monitor the suicide risk. A trained study coordinator will administer the questionnaire to assess in real-time the participant’s risk of suicide and make appropriate referrals to mental health services if necessary. If participants state or demonstrate active suicidal ideations, standard clinic procedures for care will be implemented. Specifically, a designated staff member from the specialized Alachua County Crisis Center will be dispatched to the clinic to assist the patient. Alternatively, arrangements will be made for the patient’s voluntary admission or, if necessary, their placement under the Baker Act at a psychiatric facility for appropriate management. Moreover, participants will receive support resources, including contact information for the Suicide and Crisis Lifeline, as well as other avenues for mental health assistance. Other forms of discomfort or risks, whether social, economic, or psychological are not anticipated.

### Auditing

The University of Florida Health Cancer Center Data Integrity and Safety Committee (UFHCC DISC) will review and monitor this protocol. The UFHCC DISC roles include: (i) assessment of progress and safety in the clinical trial; (ii) evaluation of reports produced by the data quality control review process; (iii) informing the sponsor about suggested actions; (iv) examination of adverse events necessitating expedited reporting according to the protocol’s specifications; and (v) notifying UFHCC-coordinated sites of adverse events that need immediate reporting and any subsequent committee recommendations for trial adjustments.

If the necessity for a protocol adjustment arises, the IRB, sponsor, study team, and participants will be notified. In the event of any concerns, the DISC will decide whether the study will require suspension.

The required level of DISC monitoring will be dictated by the risk level determined by SRMC CCPSP. Higher-risk trials usually require heightened supervision.

## Electronic Supplementary Material

Below is the link to the electronic supplementary material.

**Supplementary Material 1**: **Additional file 1.pdf**: Fillable-SPIRIT-Outcomes-2022-Checklist-with-SPIRIT-2013.

**Supplementary Material 2**: **Additional file 2.pdf**: Includes all the questionnaires that will be used for data collection.

**Supplementary Material 3**: **Additional file 3.pdf**: Informed Consent form.

**Supplementary Material 4**: **Additional file 4.pdf**: Handling of biological specimens.

## Data Availability

No datasets were generated or analysed during the current study.

## Declarations

### Minimizing potential risk to confidentiality

The study staff are extensively trained in research procedures aimed at safeguarding participants’ privacy and are accustomed to upholding confidentiality in all their activities. Additionally, all team members will complete a human subject training course before the study commences. Any reported or suspected privacy concerns will promptly be communicated to the PI.

Multiple measures are implemented to protect the identities of participants. Data files will be coded, and numerical identification codes will be assigned to participants to prevent any potential identification. Informed consent documents will be kept in locked filing cabinets behind closed doors, accessible only to authorized study personnel. Information that includes identifiers will be maintained at a separate site from the study data. Furthermore, the data storage facility will have restricted, key-controlled access, adding an extra layer of security. Data collected during the study will be entered into the HIPAA-compliant REDCap application.

### Post-trial and ancillary care

No significant adverse events are anticipated from this study; therefore, participants who may experience harm during the trial will not receive compensation.

### Dissemination policy

All findings will be documented in ClinicalTrials.gov. The results of the trial will be distributed through academic journals that are both open-access and peer-reviewed to ensure broad accessibility will be published in open-access scholarly journals to ensure broad accessibility. The full protocol, complete statistical code and participant-level dataset can be granted via the corresponding author. Additionally, the results will be shared with the public through poster and conference presentations.

### Protocol version number

0002.0
