# Supplementary material for: The potential of AB-free kava in enabling tobacco cessation via management of abstinence-related stress and insomnia: study protocol for a randomized clinical trial
Source: BMC Complement Med Ther. 2024 Dec 21;24:422. doi: 10.1186/s12906-024-04722-9 (PMC11662815; doi:10.1186/s12906-024-04722-9)
Supplement: Supplementary file 2 — Supplementary Material 2: Additional file 2.pdf: Includes all the questionnaires that will be used for data collection. [file 12906_2024_4722_MOESM2_ESM.pdf]

PROPOSED MEASURES FOR TOBACCO USE, CRAVING, ADDICTION, STRESS, AND INSOMNIA

**Self-Reported Measures of Smoking Cigarettes**

| ✓ Instructions for Filling Out the Timeline Cigarette Use Calendar |
| --- |

To help us evaluate your cigarette use, we need to get an idea of what your smoking was like in the past________________ days. To do this, we would like you to fill out the attached calendar.
Filling out the calendar is not hard! Try to be as accurate as possible.

We recognize you won't have perfect recall. That's **OKAY**.

**WHAT TO FILL IN**
The idea is to record how many cigarettes you smoked for each day on the calendar.
On days when you did not smoke cigarettes, not even one, you should write a "0."
It's important that something is written for every day, even if it is a 0".

**YOUR BEST ESTIMATE**
We realize it isn't easy to recall things with 100% accuracy.
If you are not sure whether you smoked 15 or 16 cigarettes or whether you smoked on a Thursday or a Friday, give it your best guess! What is important is that 15 or 16 cigarettes is very different from 1 cigarette. The goal is to get a sense of how frequently you smoked, how much you smoked, and your patterns of smoking.

**HELPFUL HINTS**

- If you have an appointment book, you can use it to help you recall your use.
- Holidays such as Thanksgiving and Christmas are marked on the calendar to help you recall your smoking. Also, think about how much you smoked on personal holidays & events such as birthdays, vacations, or parties.
- If you have regular patterns to your smoking, you can use these to help you recall your use.
  For example, some people may only smoke during social situations.

**COMPLETING THE CALENDAR**
A blank calendar is attached. Write in the number of cigarettes you smoked on each day.
The time period we are talking about on the calendar is from______________ to ___________________.
In estimating the number of cigarettes you smoked, be as accurate as possible.

**DOUBLE CHECK THAT ALL DAYS ARE FILLED IN BEFORE RETURNING THE CALENDAR.**
Before you start look at the **SAMPLE CALENDAR**.

**Fagerstrom Test for Nicotine Dependence**

1. How soon after you wake up do/did you smoke your first cigarette?

   1[ ]Within 5 minutes [3 points]

   2 [ ]6-30 minutes [2 points]

   3 [ ]31-60 minutes [1 point]

   4 [ ]After 60 minutes [0 points]

2. Do/Did you find it difficult to refrain from smoking in places where it is forbidden, e.g.,in church, at the library, in a cinema, etc.?

   1 [ ]Yes [1 point]

   2[ ]No [0 points]

3. Which cigarette would you hate most to give up?

   1 [ ]The first in the morning [1 point]

   2[ ]All others [0 points]

4. How many cigarettes per day do/did you smoke?

   1[ ]10 or less [0 points]

   2[ ]11-20 [1 point]

   3[ ]21-30 [2 points]

   4[ ]31 or more [3 points]

5. Do/did you smoke more frequently during the first hours after waking than during therest of the day?

   1 [ ]Yes [1 point]

   2[ ]No [0 points]

6. Do/did you smoke if you are so ill that you are in bed most of the day?

   1 [ ]Yes [1 point]

   2[ ]No [0 points]

**Modified Cigarette Evaluation Questionnaire**

**If you have smoked since you last completed this questionnaire, please mark the number that best represents how smoking made you feel (1—not at all, 2—very little, 3—a little, 4—moderately, 5—a lot, 6—quite a lot, 7—extremely).**

| | 1 | 2 | 3 | 4 | 5 | 6 | 7 |
|---|---|---|---|---|---|---|---|
| Was smoking satisfying? | [ ] | [ ] | [ ] | [ ] | [ ] | [ ] | [ ] |
| Did cigarettes taste good? | [ ] | [ ] | [ ] | [ ] | [ ] | [ ] | [ ] |
| Did you enjoy the sensations in your throat and chest? | [ ] | [ ] | [ ] | [ ] | [ ] | [ ] | [ ] |
| Did smoking calm you down? | [ ] | [ ] | [ ] | [ ] | [ ] | [ ] | [ ] |
| Did smoking make you feel more awake? | [ ] | [ ] | [ ] | [ ] | [ ] | [ ] | [ ] |
| Did smoking make you feel less irritable? | [ ] | [ ] | [ ] | [ ] | [ ] | [ ] | [ ] |
| Did smoking help you concentrate? | [ ] | [ ] | [ ] | [ ] | [ ] | [ ] | [ ] |
| Did smoking reduce your hunger for food? | [ ] | [ ] | [ ] | [ ] | [ ] | [ ] | [ ] |
| Did smoking make you dizzy? | [ ] | [ ] | [ ] | [ ] | [ ] | [ ] | [ ] |
| Did smoking | [ ] | [ ] | [ ] | [ ] | [ ] | [ ] | [ ] |

| | | | | | | | |
|---|---|---|---|---|---|---|---|
| make you nauseous? | | | | | | | |
| Did smoking immediately relieve your craving for a cigarette? | [ ] | [ ] | [ ] | [ ] | [ ] | [ ] | [ ] |
| Did you enjoy smoking? | [ ] | [ ] | [ ] | [ ] | [ ] | [ ] | [ ] |

**Brief Questionnaire on Smoking Urges**

*Indicate how much you agree or disagree with each of the following statements by placing a single checkmark (like this: [✓]) by a number ranging from 1 (strongly disagree) to 7 (strongly agree). The closer you place your checkmark to one end or the other indicates the strength of your disagreement or agreement. Please complete every item. We are interested in how you are thinking or feeling* ***right now*** *as you are filling out this questionnaire.*

| | 1 Strongly Disagree | 2 | 3 | 4 | 5 | 6 | 7 Strongly Agree |
|---|---|---|---|---|---|---|---|
| 1. I have a desire for a cigarette right now. | [ ] | [ ] | [ ] | [ ] | [ ] | [ ] | [ ] |
| 2. Nothing would be better than smoking a cigarette right now. | [ ] | [ ] | [ ] | [ ] | [ ] | [ ] | [ ] |
| 3. If it were possible, I probably would smoke right now. | [ ] | [ ] | [ ] | [ ] | [ ] | [ ] | [ ] |
| 4. I could control things better right now if I could smoke. | [ ] | [ ] | [ ] | [ ] | [ ] | [ ] | [ ] |
| 5. All I want right now is a cigarette. | [ ] | [ ] | [ ] | [ ] | [ ] | [ ] | [ ] |
| 6. I have an uge for a cigarette. | [ ] | [ ] | [ ] | [ ] | [ ] | [ ] | [ ] |

| | | | | | | | |
|---|---|---|---|---|---|---|---|
| 7. A cigarette would taste good now. | [ ] | [ ] | [ ] | [ ] | [ ] | [ ] | [ ] |
| 8. I would do almost anything for a cigarette now. | [ ] | [ ] | [ ] | [ ] | [ ] | [ ] | [ ] |
| 9. Smoking would make me less depressed. | [ ] | [ ] | [ ] | [ ] | [ ] | [ ] | [ ] |
| 10. I'm going to smoke as soon as possible. | [ ] | [ ] | [ ] | [ ] | [ ] | [ ] | [ ] |

# Perceived Stress Scale

**For each question choose from the following alternatives:**
**0 - never  1 - almost never  2 - sometimes  3 - fairly often  4 - very often**

_________ l. In the last month, how often have you been upset because of something that happened unexpectedly?

_________ 2. In the last month, how often have you felt that you were unable to control theimportant things in your life?

_________ 3. In the last month, how often have you felt nervous and stressed?

_________ 4. In the last month, how often have you felt confident about your ability to handle your personal problems?

_________ 5. In the last month, how often have you felt that things were going your way?

_________ 6. In the last month, how often have you found that you could not cope withall the things that you had to do?

_________ 7. In the last month, how often have you been able to control irritations inyour life?

_________ 8. In the last month, how often have you felt that you were on top of things?

_________ 9. In the last month, how often have you been angered because of things thathappened that were outside of your control?

_________ 10. In the last month, how often have you felt difficulties were piling up so high that you could not overcome them?

**Insomnia Severity Scale**

The Insomnia Severity Index has seven questions. The seven answers are added up to get a total score. When you haveyour total score, look at the 'Guidelines for Scoring/Interpretation' below to see where your sleep difficulty fits.

For each question, please CIRCLE the number that best describes your answer.

*Please rate the CURRENT (i.e. LAST 2 WEEKS) SEVERITY of your insomnia problem(s).*

| Insomnia Problem | None | Mild | Moderate | Severe | Very Severe |
|---|---|---|---|---|---|
| 1. Difficulty falling asleep | 0 | 1 | 2 | 3 | 4 |
| 2. Difficulty staying asleep | 0 | 1 | 2 | 3 | 4 |
| 3. Problems waking up too early | 0 | 1 | 2 | 3 | 4 |

4. How SATISFIED/DISSATISFIED are you with your CURRENT sleep pattern?

| Very Satisfied | Satisfied | Moderately Satisfied | Dissatisfied | Very Dissatisfied |
|---|---|---|---|---|
| 0 | 1 | 2 | 3 | 4 |

5. How NOTICEABLE to others do you think your sleep problem is in terms of impairing the quality of your life?

| Not at all Noticeable | A Little | Somewhat | Much | Very Much Noticeable |
|---|---|---|---|---|
| 0 | 1 | 2 | 3 | 4 |

6. How WORRIED/DISTRESSED are you about your current sleep problem?

| Not at all Worried | A Little | Somewhat | Much | Very Much Worried |
|---|---|---|---|---|
| 0 | 1 | 2 | 3 | 4 |

7. To what extent do you consider your sleep problem to INTERFERE with your daily functioning (e.g. daytime fatigue, mood, ability to function at work/daily chores, concentration, memory, mood, etc.) CURRENTLY?

| Not at all Interfering | A Little | Somewhat | Much | Very Much Interfering |
|---|---|---|---|---|
| 0 | 1 | 2 | 3 | 4 |

**Guidelines for Scoring/Interpretation:**

Add the scores for all seven items (questions 1 + 2 + 3 + 4 + 5 +6 + 7) =___

your total scoreTotal score categories:

0–7 = No clinically significant insomnia

8–14 = Subthreshold insomnia

15–21 = Clinical insomnia (moderate severity)22–28 = Clinical insomnia (severe)

*Used via courtesy of www.myhealth.va.gov with permission from Charles M. Morin, Ph.D., Université Laval*

**Ask Suicide-Screening Questions (ASQ)**

NIMH TOOLKIT

asQ Ask Suicide-Screening Questions

# Suicide Risk Screening Tool

## Ask the patient:

1. In the past few weeks, have you wished you were dead? ❍ Yes ❍ No
2. In the past few weeks, have you felt that you or your family would be better off if you were dead? ❍ Yes ❍ No
3. In the past week, have you been having thoughts about killing yourself? ❍ Yes ❍ No
4. Have you ever tried to kill yourself? ❍ Yes ❍ No

   If yes, how? ______________________________

   ______________________________

   ______________________________

   When? ______________________________

   ______________________________

*If the patient answers* **Yes** *to any of the above, ask the following acuity question:*

5. Are you having thoughts of killing yourself right now? ❍ Yes ❍ No

   If yes, please describe: ______________________________

## Next steps:

- If patient answers "No" to all questions 1 through 4, screening is complete (not necessary to ask question #5). No intervention is necessary (**Note: Clinical judgment can always override a negative screen*).
- **If patient answers "Yes" to any of questions 1 through 4, or refuses to answer, they are considered a positive screen. Ask question #5 to assess acuity:**
  - ☐ "Yes" to question #5 = **acute positive screen** (imminent risk identified)
    - **Patient requires a STAT safety/full mental health evaluation. Patient cannot leave until evaluated for safety.**
    - Keep patient in sight. Remove all dangerous objects from room. Alert physician or clinician responsible for patient's care.
  - ☐ **"No"** to question #5 = **non-acute positive screen** (potential risk identified)
    - **Patient requires a brief suicide safety assessment to determine if a full mental health evaluation is needed. Patient cannot leave until evaluated for safety.**
    - Alert physician or clinician responsible for patient's care.

## Provide resources to all patients

- 24/7 National Suicide Prevention Lifeline 1-800-273-TALK (8255) En Español: 1-888-628-9454
- 24/7 Crisis Text Line: Text "HOME" to 741-741
