# Supplementary material for: The potential of AB-free kava in enabling tobacco cessation via management of abstinence-related stress and insomnia: study protocol for a randomized clinical trial
Source: BMC Complement Med Ther. 2024 Dec 21;24:422. doi: 10.1186/s12906-024-04722-9 (PMC11662815; doi:10.1186/s12906-024-04722-9)
Supplement: Supplementary file 3 — Supplementary Material 3: Additional file 3.pdf: Informed Consent form. [file 12906_2024_4722_MOESM3_ESM.pdf]

Appendix 1: Informed Consent

UF Institutional Review Board
UNIVERSITY of FLORIDA

INFORMED CONSENT FORM

to Participate in Research, and

AUTHORIZATION

to Collect, Use, and Disclose Protected Health Information (PHI)

INTRODUCTION

Name of person seeking your consent:____

Place of employment & position:____

GENERAL INFORMATION ABOUT THIS STUDY

1. Name of Participant ("Study Subject")

____

2. What is the title of this research study (this "Research Study")?

The potential of kava in enabling tobacco cessation - its holistic effects in managing stress and insomnia associated with abstinence

3. Whom do you call if you have questions about this Research Study (the "Study Team")?

Principal Investigator: Ramzi Salloum, Ph.D, 352-294-4997

Study Coordinator(s): A'lexus Brown, 352-273-7345

Melissa Bou Malham, 352-294-5892

4. Who is paying for this Research Study?

The sponsor of this study is National Center for Complementary and Integrative Health (NCCIH).

5. In general, what do you need to know about this Research Study?

Agreeing to become involved in any research is always voluntary. By signing this form, you are not waiving any of your legal rights. If you decide not to participate in this research, you will not be penalized in any way and you will not lose any benefits to which you are entitled. If you have questions about your rights as a research subject, please call the University of Florida Institutional Review Board (IRB) office at (352) 273-9600.

a) In general, what is the purpose of the research? How long will you be involved?

Tobacco use is the leading cause of numerous preventable diseases. It’s estimated that about 70% of adult smokers want to stop smoking and over 50% of them try to do so annually. AB-free Kava (a specialized Kava supplement) has the potential to be a promising intervention in improving rates of successful tobacco cessation. This study seeks to explore variables that would help or hinder a tobacco cessation strategy using AB-free Kava. Additionally, it aims to determine if AB-free Kava can help lessen the negative biological effect tobacco has on the body. The study drug may have the potential to minimize side effects of tobacco withdrawal such as stress and insomnia.

The information collected from this trial will help to assess how viable AB-free Kava is as an effective tool in tobacco cessation. Tobacco use is the leading cause of many preventable diseases. Unfortunately, around 16% of US adults continue to smoke cigarettes due to its addictive nature and the limited success of current cessation strategies, partly because these cessation strategies have various adverse effects, such as suicide risk, anxiety, and insomnia. Therefore, there is an unmet and urgent need for novel interventions to improve the success of tobacco cessation. Kava is a traditional beverage consumed daily by residents of the South Pacific Islands to promote relaxation, to socialize and to improve the quality of sleep. Kava has

also been available as a dietary supplement in the US for several decades to support calm and relaxation. This study will use AB-free kava which is a new formulation that is expected to have a better safety profile than other kava supplements. This study will document AB-free kava use compliance, its effects on smoking, stress, and sleep associated behaviors. This study will also look into the various reasons why people will enroll in the study and their willingness to participate in the study. You may be involved in this research for 12 weeks.

b) What is involved with your participation, and what are the procedures to be followed in the research?

This trial will enroll active tobacco smokers who have a desire to quit using tobacco. Should you choose to participate, you will be randomly assigned to 1 of 2 groups. This randomization process is similar to blindly drawing a number from a hat. The study is "blinded" meaning that neither you nor your doctor will know what group you have been assigned to during the study. You could be assigned to one of the following groups:

- You will receive AB-free Kava supplements
- You will receive placebo supplements that will have no AB-free kava

The table below shows a general overview of what you can expect from each of your 6 study visits (described as Visit 0 - Visit 6).

Table 1. Study Visits

	Visit 0<br>Screening	Visit 1<br>Week 0	Visit 2<br>Week 1	Visit 3<br>Week 2	Visit 4<br>Week 4	Visit 5<br>Week 8	Visit 6<br>Week 12
**$50 Gift Card**		X	X	X	X	X	X
**Brief Physical Exam**	X				X		
**CO Breath Test**	X	X	X	X	X	X	X

**Questionnaires**	X	X	X	X	X	X	X
**Blood Sample**	X	X	X	X	X	X	X
**Urine Sample**		X	X	X	X	X	X
**Receive Study Pills**		X	X	X			
**Pregnancy Test (if applicable)**	X						
**Exit Interview**							X
**Post-Treatment Interview (OPTIONAL with an additional $25 Gift Card if participate)**					X		

For a more detailed description of what your participation in this study will include, please refer to Section 7.

c) What are the likely risks or discomforts to you?

Possible discomforts and risks from taking the AB-free kava supplement are expected to be minimal. The AB-free kava utilized in this study is expected to have an improved safety profile above other kava supplements. Nonetheless, this AB-Free Kava supplement still has the potential to cause side effects.

AB-Free kava use may produce feelings of relaxation or drowsiness, such that driving or operation of heavy machinery should be avoided within 2-hours after consumption until you know how it may affect you, if any effect at all. Other potential side effects include digestive upset, headache, and dizziness.

This study includes an Ask Suicide-Screening Questions form (ASQ) to assess suicide risk given kava’s potential effects on the central nervous system. This form is required by the FDA as an added safety measure, although such risks have not been observed in studies of previous kava use. Kava use has been suggested to potentially increase the risk of liver toxicity; therefore you

are asked to abstain from using over-the-counter acetaminophen (e.g. Tylenol®, etc.) during the trial period which may put added burden on your liver. You will be provided a list of medications that contain acetaminophen for your reference. You will also be asked to limit your alcohol consumption to, at most, one drink a day while on this study. Excessive alcohol consumption may result in withdrawal from the study (greater than one drink per day). Subjects with pre-existing liver conditions will be excluded from participation and liver function will be monitored throughout the study with blood tests as an added measure of safety. If there are abnormal results found in these tests, you will be notified immediately and retested in the next 48 – 72 hours, or completely discontinued from the study if significant and/or accompanied by other symptoms.

The risks of drawing blood from a vein include discomfort at the site of puncture, possible bruising and swelling around the puncture site, rarely an infection, and, uncommonly, faintness from the procedure.

d) What are the likely benefits to you or to others from the research?

AB-free kava may or may not provide those randomized to either of the AB-free kava groups a reduction in the urge to smoke and use tobacco. It may or may not also reduce damage caused by tobacco carcinogens and increase the clearance of NNAL, a known carcinogen in lung cancer, and thereby reduce lung cancer risk.

e) What are the appropriate alternative procedures or courses of treatment, if any, that might be helpful to you?

There are other smoking cessation programs, such as government programs available through Tobacco Free Florida, you may wish to try if you wish to stop smoking but do not wish to participate in this study. Your physician can review these with you if you wish.

A description of this clinical trial will be available on http://www.ClinicalTrials.gov(47,48) as required by U.S. Law. This website will not include information that can identify you. At most, the website will include a summary of the results. You can search this website at any time.

Additional and more detailed information is provided within the remainder of this Informed Consent form. Please read before deciding if you wish to participate in this study.

WHAT CAN YOU EXPECT IF YOU PARTICIPATE IN THIS STUDY?

6. What will be done as part of your normal clinical care (even if you did not participate in this Research Study)?

There are other smoking cessation programs, such as government programs available through Tobacco Free Florida, you may wish to try if you wish to stop smoking but do not wish to participate in this study. Your physician can review these with you if you wish.

7. What will be done only because you are in this Research Study?

You may need to have the following exams, tests or procedures to find out if you can be in the study. If you had some of these tests done recently, they may not need to be repeated. This will be up to your study doctor.

- Medical history
- Physical exam with vital signs
- Blood tests to evaluate your blood counts and blood chemistry
- Urine Sample collected
- Pregnancy testing (if applicable)
- Current medications review
- CO Breath Test

If you are eligible and decide to take part in this study, you will be randomly assigned (much like a number drawn from a hat) to receive either placebo or AB-free kava. Pills will be administered at Visit 1, Visit 2, and Visit 3. Your participation will involve taking one capsule three times daily at approximately 8 AM, 1PM, and 6PM for four weeks.

A placebo is a substance that looks like and is given in the same way as an experimental treatment but contains no medicine, for example a sugar pill. A placebo is used in research studies to show what effect a treatment has compared with taking nothing at all. If you are assigned to receive placebo, you will not receive the benefits of the AB-free Kava, if there are any, nor will you be exposed to its risks, which are described below under "What are the possible discomforts and risks?" Studies have shown, however, that about 1 in 3 persons who take a placebo do improve, if only for a short time. You and the physician and other persons doing the study will not know whether you are receiving placebo or the study drug, but that information is available if it is needed after the study. Also, you will have a 2 in 3 (~66%) chance of receiving AB-free kava and a 1 in 3 (~33%) chance of receiving placebo. In the remainder of the description of what will be done, both the AB-free kava and the placebo will be called "study treatment."

The dose of AB-free kava (if you are assigned to the full-dose AB-free Kava group), will be 75 mg kavalactones per capsule. If you are assigned to the half-dose group, then it will be 37.5 mg kavalactones per capsule. Concluding the study’s treatment period, you will have two additional follow-up visits. Once enrolled, you will have a total of 6 visits over 12 weeks, which are needed. Each visit, while you are on the study, will last about 30 to 60 minutes. Please let the study team know if you do not think you can attend all 6 research visits.

At your initial screening visit (Visit 0), you will receive a GT3X+ wearable device that will measure your physical activity and sleep. These sensors will record your data while you have the GT3X+ wearable device on. As part of this study, you will wear the device on your wrist for 4 days after your four treatment visits (Visit 1 – Visit 4). You will be expected to return the wearable device to a member of the study coordinator at each visit for the next 4 weeks (Visit 1-4). The study coordinator will download your data, recharge the device, and return it to you in the same visit. This will be repeated for 4 weeks so that four sets of measures can be collected. The Research Coordinator will provide written instructions and demonstrate how to correctly wear the device. At the end of Visit 4, this device will be returned to the study coordinator.

At your Visit 1, Visit 2, and Visit 3 appointments, you will receive pills that will either be placebo capsules, full-dose AB-free Kava capsules, or half-dose AB-free Kava capsules, that will last you till the next visit. The capsules will be given to you by the Study Coordinator.

During each of the six visits, you will undergo lab tests of your blood and urine, a CO breath test, and complete questionnaires about your smoking, behaviors, addiction (i.e. alcohol use), urges, stress, insomnia, and suicidality.

- For each blood draw, a study coordinator will collect 10 ml (approximately 2 teaspoonfuls) of blood. After your blood sample is drawn a study coordinator member will contact you with the results within 24 hours of your labs being done and let you know to either continue taking the pills or stop taking the pills. During this 24-hour period, please take the pills as instructed. If you have not heard from a study team member within 24 hours of your labs being done, please stop taking the pills until you are contacted. An additional 10 ml (approximately 2 teaspoonfuls) of blood will be collected during each visit for research testing.

- For the urine collection, you will be given a urine container (like a jug) to collect all of the urine that you void for a 24-hour period before your next visit. For example, when you receive an empty urine container at your Screening Visit (Week 0) appointment to take home, you will collect urine 24 hours before your Week 1 appointment, fill the container, and bring it in with you at that time to return to the study coordinator.
- The CO breath test will require you to exhale into a breathalyzer device to measure your Carbon Monoxide (CO) levels.

If any identifiable information or identifiable biospecimens were collected as part of this research, it is possible that your research information or specimens, with all personally identifiable information removed, could be used for future research studies. There is a possibility it could be distributed to another investigator for future research studies without additional informed consent from you or your legally authorized representative.

At the end of the study (Visit 6) or if you voluntarily choose to leave the study sooner than that, an exit interview will be performed with open-ended questions to obtain feedback about the trial.

At Visit 3 you will be offered an opportunity to participate in a separate in-depth, after-treatment interviews at Visit 4 to help identify factors that either helped or hindered your experiences in the trial. This interview will be 15 – 60 minutes with an additional $25 Gift Card. You will be audio-recorded during these interviews. If you choose to not participate in the post-treatment interview, then you will not receive the $25.

If at any point in this study you no longer want to participate, you may freely withdraw with no consequences to your current or future health care needs. If you have any questions now or at

any time during this Research Study, please contact one of the Research Team members listed in question 3 of this form.

8. What identifiable health information will be collected about you and how will it be used?

The Research Team will collect demographic information, results of physical exams, blood tests, and other diagnostic and medical procedures, as well as medical history.

The Research Team may collect this information from other healthcare providers, such as laboratories, which are a part of this research, as well as healthcare providers that are not part of this research (other doctors, hospitals or clinics). Other professionals at the University of Florida or Shands Hospital who provide study-related care, and the University of Florida Institutional Review Board (IRB), may also collect your health information.

The Research Team listed in question 3 above will use or share your health information as described below to carry out this research study.

9. With whom will this health information be shared?

This health information may be shared with:

- the study sponsor (listed in Question 4 of this form);
- United States governmental agencies which are responsible for overseeing research, such as the Food and Drug Administration, the Department of Health and Human Services, and the Office of Human Research Protections;
- government agencies which are responsible for overseeing public health concerns, such as the Centers for Disease Control and federal, state and local health departments,
- Your insurance company for purposes of obtaining payment, and
- the IRB that reviewed this Research Study and ensures your rights as a Study Subject are protected.

Otherwise, your identifiable health information will not be shared without your permission unless required by law or a court order. Once your health information is shared with those listed above, it is possible that they could share it without your permission because it would no longer be protected by the federal privacy law.

10. How long will you be in this Research Study?

Your participation in this study is expected to last approximately 12 weeks.

This Authorization to use and share your health information expires at the end of the study, unless you revoke it (take it back) sooner.

11. How many people are expected to take part in this Research Study?

Seventy-six (76) subjects are expected to be deemed eligible and take part in this study.

WHAT ARE THE RISKS AND BENEFITS OF THIS STUDY AND WHAT ARE YOUR OPTIONS?

12. What are the possible discomforts and risks from taking part in this Research Study?

The risks of drawing blood from a vein include discomfort at the site of puncture; possible bruising and swelling around the puncture site; rarely an infection; and, uncommonly, faintness from the procedure.

Possible discomforts and risks from taking a kava supplement are minimal, based on previous data from human studies. AB-free kava will be used in the study, which is expected to have an improved safety profile above other kava supplements. Because there is a potential for AB-free kava to affect the function of the liver, subjects with liver conditions will be excluded and we will monitor liver function throughout the study with safety blood tests. You will also be asked to limit one drink per day and not take acetaminophen. You will be provided a list of medications containing acetaminophen. If there are negative effects found in these tests, you will be notified

immediately and retested in the next 48 – 72 hours, or completely discontinued from the study if severe and/or accompanied by other symptoms. At the same time, AB-free kava use may cause sedation such that driving or operation of heavy machinery is not recommended. Additional attention is needed for driving within 2 hours after AB-free kava use, until you know how AB-free kava will affect you. Other side effects that could occur include digestive upset, headache, and dizziness.

Subjects should avoid becoming pregnant while on study and agree to practice acceptable methods of birth control to avoid pregnancy (birth control pills, injection, IUD, diaphragm, condoms, or cervical cap). A pregnancy test will be performed during screening for applicable subjects, but if you think you may be pregnant during the course of study participation, notify the study team listed in item 3 of this document.

The Ask Suicide-Screening Questions form (ASQ) has been built into the study to assess the suicide risk given kava's potential neurological functions per FDA's suggestion, however, the risk of kava induced suicide is minimal, if any, based on previous research with no report(s) of such suicide risks in clinical trials. This is a research study that involves questions related to sensitive topics. As researchers, we do not provide mental health services. However, we want to provide you with contact information for available resources, should you decide you need assistance at any time. Here are some numbers for available resources; UF psychiatry clinical sites (352) 265-4357., the Alachua county Crisis Center (352) 264-6789, and the Suicide and Crisis Lifeline 988 number. Should an emergency arise in clinic (statement of demonstration of active suicide ideation) standard of care clinic procedures will be followed. This involves calling Alachua County Crisis Center to have an in-clinic evaluation or sending the patient directly to a

psychiatric facility. Please ask the study coordinator if you would like more information on these clinic procedures.

Other possible risks to you may include emotional discomfort at responding to some survey questions. However, you are not required to answer any question you wish to skip.

To help us protect your privacy, we have obtained a Certificate of Confidentiality from the National Institutes of Health. With this Certificate, the researchers cannot be forced to disclose information that may identify you, even by a court subpoena, in any federal, state, or local civil, criminal, administrative, legislative, or other proceedings. The researchers will use the Certificate to resist any demands for information that would identify you, except as explained below.

The Certificate cannot be used to resist a demand for information from personnel of the United States Government that is used for auditing or evaluation of federally funded projects or for information that must be disclosed in order to meet the requirements of the federal Food and Drug Administration (FDA).

You have been informed that a Certificate of Confidentiality does not prevent you from voluntarily releasing information about yourself or your involvement in this research. If an insurer, employer, or other person obtains your written consent to receive research information, then the researchers may not use the Certificate to withhold that information. That is, if you give written consent for the release of information, we cannot withhold that information and we cannot hold responsibility for how that person may use your information.

The Certificate of Confidentiality does not prevent the researchers from disclosing voluntarily, without your consent, information that would identify you as a participant in the research project under the following circumstances. If we learn about child abuse, elder abuse, or intent to harm yourself or others, we will report that information to appropriate authorities.

This Research Study may also include risks that are unknown at this time.

Please note, participating in more than one research study or project may further increase the risks to you. If you are already enrolled in a research study, please inform one of the Research Team members listed in question 3 of this form or the person reviewing this consent with you before enrolling in this or any other research study or project.

During the study, the Research Team will notify you of new information that may become available and might affect your decision to remain in the study.

The University of Florida is required by law to protect your health information. Your health information will be stored in locked filing cabinets or on computer servers with secure passwords, or encrypted electronic storage devices, as required by University policy. However, there is a slight risk that information about you could be released inappropriately or accidentally. Depending on the type of information, a release could upset or embarrass you, or possibly affect your ability to get insurance or a job.

If you wish to discuss the information above or any discomforts you may experience, please ask questions now or call one of the Research Team members listed in question 3 in this form.

13a. What are the potential benefits to you for taking part in this Research Study?

AB-free kava may or may not provide those randomized to the AB-free kava group a reduction in the urge to smoke and tobacco use. It may or may not also reduce damage caused by tobacco carcinogens and increase the clearance of NNAL, a known carcinogen in lung cancer, and thereby reduce lung cancer risk.

13b. How could others possibly benefit from this Research Study?

If AB-free kava supplementation is able to facilitate tobacco cessation and is safe, it may help inform smoking cessation programs for other healthy smokers.

13c. How could the Research Team members benefit from this Research Study?

In general, presenting research results helps the career of a researcher. Therefore, the Research Team listed in question 3 of this form may benefit if the results of this Research Study are presented at scientific meetings or in scientific journals. The results may also lead to new knowledge and experience that will help expand and initiate similar studies in the future.

Dr. Chengguo Xing, Co-Investigator, might benefit financially from this study. Specifically, Dr. Xing is the inventor of the new treatment being studied, and Dr. Xing has an investment in Kuality Herbceutics, such as stock. Kuality Herbceutics is a start-up company that was created to make AB-free kava widely available. Research studies, like the one you are thinking of joining, are done to determine whether the new treatment is safe and effective. If research shows the new treatment is safe and effective, Dr. Xing may receive a part of the profits from any sales. In addition, the amount of money Dr. Xing’s investment is worth might be affected by the results of this study. This means that Dr. Xing could gain or lose money depending on the results of this study. The Institutional Review Board and the University of Florida have reviewed the possibility of the financial benefit. They believe that the possible financial benefit to the person leading the research is not likely to affect your safety and/or the scientific quality of the study. If you would like more information, please ask the researchers or study coordinator.

13d. Will you be allowed to see the research information collected about you for this Research Study?

You may not be allowed to see the research information collected about you for this Research Study, including the research information in your medical record, until after the study is completed. When this Research Study is over, you will be allowed to see any research information collected and placed in your medical record.

14. What other choices do you have if you do not want to be in this study?

You may choose not to participate in this study. There are other smoking cessation programs you may wish to try if you wish to stop smoking but do not wish to participate in this study, such as government programs available through Tobacco Free Florida. Your physician can review these with you if you wish.

You may also refuse to authorize the use of your health information, but if you refuse, you may not be allowed to be in this research study or receive any research-related treatment that is only available in this research study. However, your decision not to sign this Authorization will not affect any other treatment you may be eligible to receive.

15a. Can you withdraw from this study?

You may withdraw your consent and stop participating in this Research Study at any time. If you do withdraw your consent, there will be no penalty to you, and you will not lose any benefits to which you are otherwise entitled. If you decide to withdraw your consent to participate in this Research Study for any reason, please contact the Research Team listed in question 3 of this form. They will tell you how to safely stop your participation.

You can also change your mind and take back this Authorization at any time by sending a written notice to the Research Team listed in question 3 of this form to let them know your decision. If you take back this Authorization, the Research Team may only use and disclose your health information already collected for this research study. No additional health information about you will be collected or disclosed to the Research Team. However, if you take back this Authorization, you may not be able to continue in this study. Please discuss this with a member of the Research Team listed in question #3.

15b. Can the Principal Investigator withdraw you from this Research Study?

You may be withdrawn from this Research Study without your consent for the following reasons:

- Your lab tests show it is not safe for you to continue, or your physician believes it is unsafe for you to continue treatment
- You are not compliant with the treatment schedule
- You are not compliant with the alcohol consumption restriction of one drink per day
- You become pregnant during the course of study treatment*

*If you believe you are pregnant or have become pregnant during the course of this study, please contact study coordinator listed in question #3 at the top of this document.

WHAT ARE THE FINANCIAL ISSUES IF YOU PARTICIPATE?

16. If you choose to take part in this Research Study, will it cost you anything?

Study Drug

The study drug, AB-free kava (full and half-dose) or placebo will be provided at no cost to you while you are participating in this study.

Study Services

No, there will be no additional costs to you or your health plan as a result of your participation in this study. The sponsor will pay for all health care costs related to your participation, including all required study items, services and procedures described in this consent form. However, if you feel you have received a bill related to this study, please contact the Principal Investigator.

If you receive any healthcare at UF Health that is not related to this study, this care will be billed as usual.

17. Will you be paid for taking part in this Research Study?

You will be paid $50 per study visit at the end of each visit for a total of up to $300.

That $300 total does not include the additional $25 from the post-treatment interview. If you attend every study visit and the post-treatment interview, then the total will be $325.

If you are paid more than $199 for taking part in this study, your name and social security number will be reported to the appropriate University employees for purposes of making and recording the payment as required by law. You are responsible for paying income taxes on any payments provided by the study. Payments to ***nonresident aliens*** must be processed through the University of Florida Payroll and Tax Services department. If the payments total $600 or more in

a calendar year, the University must report the amount you received to the Internal Revenue Service (IRS). The IRS is not provided with the study name or its purpose. If you have questions about the collection and use of your Social Security Number, please visit: http://privacy.ufl.edu/SSNPrivacy.html.

Your payment for participation in this research study is handled through the University of Florida's Research Participant Payments (RPP) Program. Your information which will include your name, address, date of birth, and SSN (depending on amount of money you are paid) is protected. Access to the (RPP) Program site is limited to certain staff with the assigned security role. You will be randomly assigned a specific identification (ID) number to protect your identity. If you have any problems regarding your payment contact the study coordinator listed in question 3.

18. What if you are injured while in this Research Study?

If you are injured as a direct result of your participation in this study, only the professional services that you receive from any University of Florida Health Science Center healthcare provider will be provided without charge. These healthcare providers include physicians, physician assistants, nurse practitioners, dentists or psychologists. Any other expenses, including Shands hospital expenses, will be billed to you or your insurance provider.

You will be responsible for any deductible, co-insurance, or co-payments. Some insurance companies may not cover costs associated with research studies or research-related injuries. Please contact your insurance company for additional information.

The Principal Investigator will determine whether your injury is related to your participation in this study.

No additional compensation is routinely offered. The Principal Investigator and others involved in this study may be University of Florida employees. As employees of the University, they are protected under state law, which limits financial recovery for negligence.

Please contact one of the Research Team members listed in question 3 of this form if you experience an injury or have questions about any discomforts that you experience while participating in this Research Study.

SIGNATURES

As an investigator or the investigator's representative, I have explained to the participant the purpose, the procedures, the possible benefits, and the risks of this Research Study; the alternative to being in the study; and how the participant's protected health information will be collected, used, and shared with others:

____ ___________________

Signature of Person Obtaining Consent and Authorization Date

You have been informed about this study's purpose, procedures, possible benefits, and risks; the alternatives to being in the study; and how your protected health information will be collected, used and shared with others. You have received a copy of this form. You have been given the opportunity to ask questions before you sign, and you have been told that you can ask questions at any time.

You voluntarily agree to participate in this study. You hereby authorize the collection, use and sharing of your protected health information as described above. By signing this form, you are not waiving any of your legal rights.

____ ___________________

Signature of Person Consenting and Authorizing Date

Consent to be Audio Recorded

You have been informed that you will have audio recorded interview(s) as part of your participation in this study. Your name or personal information will not be identified on the audio recordings and confidentiality will be strictly maintained. However, when these audio recording(s) are shown or heard, others may be able to identify you.

The Principal Investigator (PI) of this study, Dr. Ramzi Salloum, or his successor, will keep the audio recording(s) in a locked cabinet, in a folder on a password protected computer server drive, or as an encrypted electronic file until transcribed. Audio files (mp3) will be downloaded immediately after the interview onto a secure UF computer. These audio recording(s) could be heard under Dr. Salloum’s direction to students, researchers, doctors, or other professionals and persons.

No identifying information will be used in any publications or presentations to protect your confidentiality. Audio files will be destroyed once analysis has been completed. Transcripts will be destroyed once all analyses are completed or within two years once the study period has ended.

By signing below, you voluntarily agree to have your study interview(s) recorded, analyzed, and then transcribed by Dr. Salloum for research purposes.

____ ____________________

Signature of Person Consenting and Authorizing Date
