# Supplementary material for: The potential of AB-free kava in enabling tobacco cessation via management of abstinence-related stress and insomnia: study protocol for a randomized clinical trial
Source: BMC Complement Med Ther. 2024 Dec 21;24:422. doi: 10.1186/s12906-024-04722-9 (PMC11662815; doi:10.1186/s12906-024-04722-9)
Supplement: Supplementary file 4 — Supplementary Material 4: Additional file 4.pdf: Handling of biological specimens. [file 12906_2024_4722_MOESM4_ESM.pdf]

Appendix 2: Biological Specimens

Blood processing:

Each visit:

a. Blood (10 mL) will be collected.
b. Within 10 minutes of collection, blood in the 10 mL purple-top tube needs to be processed.
    - https://www.youtube.com/watch?v=TKxJ4eYkt4M
    - Blood will be separated into its respective components (plasma, buffy coat, and RBCs) using a centrifuge.
    - Spin the blood tubes to further define the buffy coat layer (1200g for 10 min).
    - Using a plastic pipette, withdraw plasma and place in a cryogenic storage vial (4 mL), leaving behind ~2-3 mm of the plasma above the buffy coat.
    - Using a plastic pipette, carefully aspirate the buffy coat from the tube, and transfer to a cryogenic storage vial (2 mL). Include ~2 mm of the RBC directly below the buffy coat boundary.
    - Using a plastic pipette, transfer RBCs into a cryogenic storage vial (4 mL).
c. Store all blood samples at -20°C. These will be transferred to the Xing lab to Xing or Lynch, which will be stored at -80°C. The sample inventory will be updated.

Supplies needed at the clinic: 10-mL purple-top tube (to be purchased at the clinic), plastic pipette (stocked at the clinic), and cryogenic storage vial (stocked at the clinic).

Urine collection and processing:

Each visit:

Collection:

a. Provide participant with one 3L collection jug (Neta SIM-B350or Fisher 82028-222), female participants additional with commode specimen collection “hat” (McKesson 16-9522-CS100), a cold pack and a cool bag (https://hotcoldbads.com). The urine jug will be labeled with the sample following the Sample Labeling Codes.

b. Instruct participant to start the collection AFTER they first wake up and urinate for the day. They will mark the time of their first void and then collect ALL their urine for 24 hrs. (i.e. if participant wakes at 9 am, they do not collect that urine, but marks 9 am as the start time. They will then collect all urine until 9 am on the following day starting with the 2nd time they urinate on the first day).

c. Participant should return the urine to the clinic the day they complete the collection. Query as to whether any voids were missed.

d. Sample should be kept cool in the cool bag with the cold pack (i.e. not kept in hot car; the cold pack should be cooled in the freezer before use) and transferred to Xing lab.

e. Sample prep for storage (to be performed in Xing lab):

    - DO NOT fill past the last gradient mark or the tubes/bottles may rupture upon freezing.
    - Invert jug several times prior to aliquoting to make sure sample is well mixed.
    - Measure the total urine volume in designated 500 mL glass graduated cylinder to the closest 10 mL.

- Make 10 aliquots of 1 mL in 2 mL cryovials (VWR – 82050-210), 5 aliquots of 15 mL in 15 mL tubes (Fisher – 05-538-59A), and 2 aliquots of 100 mL in 125 mL polypropylene bottles (VWR 414004-124).
- Store at -80°C Freezer

Supplies needed at the clinic: 3-L urine collection container (stocked at the clinic), urine hats (stocked at the clinic), cool bag (stocked at the clinic), and cold pack (stocked at the clinic).
